# Constructing Indices of Rural Living Standards in Northwestern Bangladesh

**DOI:** 10.3329/jhpn.v28i5.6160

**Published:** 2010-10

**Authors:** Snaebjorn Gunnsteinsson, Alain B. Labrique, Keith P. West, Parul Christian, Sucheta Mehra, Abu Ahmed Shamim, Mahbubur Rashid, Joanne Katz, Rolf D.W. Klemm

**Affiliations:** ^1^ JiVitA Project, House 63, Road 3, Karanipara, Rangpur, Bangladesh; ^2^ Department of Economics, Yale University, New Haven, CT 06520, USA; ^3^ Global Disease Epidemiology and Control; ^4^ Center for Human Nutrition, Department of International Health, Johns Hopkins Bloomberg School of Public Health, 615 North Wolfe Street, Baltimore, MD 21205, USA

**Keywords:** Health, Living standards, Socioeconomic status, Wealth index, Bangladesh

## Abstract

This study aimed to construct indices of living standards in rural Bangladesh that could be useful to study health outcomes or identify target populations for poverty-alleviation programmes. The indices were constructed using principal component analysis of data on household assets and house construction materials. Their robustness and use was tested and found to be internally consistent and correlated with maternal and infant health, nutritional and demographic indicators, and infant mortality. Indices derived from 9 or 10 household asset variables performed well; little was gained by adding more variables but problems emerged if fewer variables were used. A ranking of the most informative assets from this rural, South Asian context is provided. Living standards consistently and significantly improved over the six-year study period. It is concluded that simple household socioeconomic data, collected under field conditions, can be used for constructing reliable and useful indices of living standards in rural South Asian communities that can assist in the assessment of health, quality of life, and capabilities of households and their members.

## INTRODUCTION

Measuring relative wealth or living standards of people in developing countries presents many challenges, especially since income data are often not available. Recent studies have addressed this problem by constructing measures based on information on household assets and dwelling characteristics using principal component analysis (PCA) (1–3). We applied this approach to household-level social and economic data and compared findings with health, nutritional status, and vital outcome data, collected during the course of a large, randomized micronutrient intervention trial, covering a substantial rural area of northwest Bangladesh. The longitudinal, population-based design, large size, and range of variables on which data were collected allowed us to directly compare household living standards and wealth indices with various nutrition and health-related characteristics usually considered to vary with socioeconomic status.

The ability to construct such asset-based indices of living standards—sometimes referred to as socioeconomic position, wealth index, or socioeconomic index—has widespread applicability since information on dwelling characteristics and durable assets (a) is available from many large studies, such as the Demographic and Health Surveys (DHS), the World Health Survey (WHS) of the World Health Organization, and the Living Standards Measurement Survey (LSMS) of the World Bank (1–2); (b) has been collected in many research studies (such as the application presented here); and (c) is often more easily and reliably collected in a developing-country setting compared to income or consumption data (1). Due to these advantages and as this approach is relatively new, having been first used by Filmer and Pritchett in 2001 (2), it is important to explore the properties of these indices and evaluate their outputs against conventional health and other indicators that are known to vary with social and economic standing across different countries and regions. One of the objectives of this research is to explore these properties using data from Bangladesh. We did not find any study applying this approach to data from a community trial but doing so allows us to relate the findings to a large number of cross-sectional and prospective health, nutrition, and demographic measures. We also explored (a) which assets, commonly assessed in field research and survey settings, yield the most information and (b) how many of such assets are needed for constructing a reliable and well-performing index.

Bollen *et al*. provided an overview of measures used for determining socioeconomic status (SES) in studies of fertility and health in developing countries and concluded that researchers have not reached a consensus on the conceptual meaning or construction of an SES indicator (4). Many researchers consider a household's consumption to be the best measure of its living standards (5). Consumption data are often not available due to the challenges inherent in ascertaining consumption reliably, and consequently, consumption-based measures might also be inappropriate when the objective is to measure household living standards over longer time periods, where multiple assessments are warranted. Constructing measures from asset rather than consumption information is also likely to be less affected by recall bias, measurement error in questions, and the effects of seasonality (1). A number of methods of varying quality exist to create indices of living standards or wealth; these are usually aggregates of a number of indicators of wealth, contextually appropriate for and adjusted to the community under study. Investigators are challenged to select an appropriate method to evaluate this important aspect of their study population that is both effective in distinguishing the spread of status within the community but that also permits wider extrapolation to other local, regional and international populations.

Bollen *et al*. have compared different indices based on assets, including a few based on estimated asset value, an index constructed as a simple sum of items owned and an index constructed using PCA (6). They found that indices that were based on the estimated asset value did not perform well and that the index constructed with PCA was superior to others as a predictor of fertility. Since the 2001 review by Bollen *et al*., a number of studies have shown asset-based indices derived using PCA to be valid and robust measures of relative living standards (1–2,6,7). Finally, studies that compared measures based on assets to those based on consumption concluded that they yield similar results (1,2,7,8).

## MATERIALS AND METHODS

### Collection of field data

Data for the study were collected as part of a large randomized, placebo-controlled community trial conducted by the JiVitA Project from 2001 to 2007 to evaluate the effects of maternal vitamin A or β-carotene supplementation on maternal, foetal and infant mortality (9). The JiVitA Project area is located in a large, contiguous rural area of Gaibandha and Rangpur districts in northwest Bangladesh; the mainly agrarian population is fairly homogeneous across a geographic area covering ∼435 sq km. During a baseline census, approximately 125,000 households (defined as a group of individuals sharing a common cooking stove) were identified, enumerated, and provided a spatial geo-coordinate (10). At the outset, a pool of 110,000 resident married women of reproductive age was enumerated, enlisted for pregnancy surveillance, and prospectively visited every five weeks by trained female staff. Pregnant women were identified by a 30-day history of amenorrhoea and a positive urine-based pregnancy test. Following informed consent, newly-pregnant women were enrolled into the trial, administered a community-allocated supplement each week, and asked to participate in a series of interviews in the home at the end of the first and third trimesters and the first six months postpartum.

At the first trimester visit, trained interview staff administered structured, pretested sets of questionnaire to elicit data on history of previous pregnancy, early pregnancy morbidity symptoms, work performed, and frequencies of dietary intake during the previous week. Household socioeconomic status was also evaluated at enrollment with respect to house, size and construction materials, land, livestock and ownership of durable assets, and occupations and education of the pregnant woman and her husband. Participants could refuse to answer any question or part of an interview. Completed sets of questionnaire were cross-checked in the field by fellow workers (peer-based verification) for errors and missing values. Trained data-entry teams entered data using a customized software with requisite range and error validation checks. As socioeconomic status variables, from which indices were derived, tend to be relatively stable, entered data that appeared inconsistent or incorrect were usually returned to the field for clarification or correction, adding to their completeness and reliability. The trial methods are discussed in greater detail elsewhere (9).

### Construction of indices

The analysis is based on socioeconomic, demographic, health- and nutrition-related data collected on a series of around 60,000 rural Bangladeshi pregnant women who were enrolled, supplemented, and followed in the above-described field trial, and nearly 7,000 additional women on whom we had data but whose follow-up period extended beyond the trial close-out date on 31 December 2006, for a total sample-size of 67,093. The R programming environment was used for statistical analysis [R Development Core Team, Vienna, Austria. (http://www.r-project.org)].

**Fig. 1.**
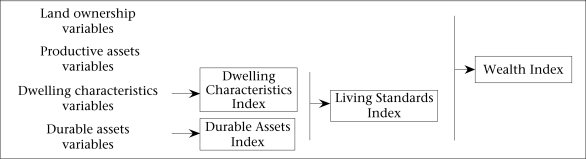
The variable groups that were used for constructing each of the indices

We used PCA and followed the methodology used in recent studies (1–3) to develop socioeconomic indices that are depicted in Figure 1. The variables chosen for analysis were divided into four categories: (a) dwelling characteristics, (b) ownership of land, (c) productive assets (other than land), and (d) durable assets (Table 1). We excluded variables for which the same answer was given by virtually every respondent or if we had reason to believe that these were weak measures of economic status. We constructed a ‘Dwelling Characteristics Index’ and a ‘Durable Assets Index’ using variables from those two categories and two composite indices using dwelling characteristics and durable assets to construct a ‘Living Standards Index’ (LSI) and by combining all four—dwelling characteristics, durable assets, ownership of land, and productive assets—into a ‘Wealth Index’ (WI) (Fig. 1).

**Table 1.** Summary of dwelling characteristics, durable assets, and living standards indices

Asset	Weight on indices		Descriptives		Mean by LSI level	
	DC	DA	LS	Mean	SD	Low	Middle	High
Walls								
No walls, thatch, grass sticks, or branches	-0.7		-0.3	0.32	0.47	0.64	0.15	0.02
Tin or wood-plank [Reference]				0.45	0.5			
Mud	0.01		-0.08	0.18	0.39	0.21	0.22	0.07
Cement	1.14		0.65	0.05	0.21	0	0.01	0.23
Roof								
No roof, thatch, or grass	-0.75		-0.3	0.12	0.33	0.27	0.03	0
Tin [Reference]				0.87	0.33			
Cement	0.5		0.16	0.01	0.08	0	0.01	0.01
Kitchen								
No separate room	-0.54		-0.24	0.33	0.47	0.57	0.22	0.06
Separate room, within house	0.11		0.03	0.11	0.31	0.09	0.12	0.11
Outside, not enclosed	0.01		-0.06	0.04	0.2	0.05	0.04	0.03
Outside home with roof [Reference]				0.52	0.50			
Toilet								
None/field/bush [Reference]				0.55	0.50			
Open/hanging latrine	-0.11		-0.01	0.01	0.11	0.01	0.02	0.01
Pit-latrine	-0.12		-0.01	0.09	0.28	0.07	0.11	0.07
Water-sealed/slap	0.68		0.35	0.35	0.48	0.05	0.41	0.85
Flush toilet	1.14		0.68	0	0.05	0	0	0.01
Electricity								
No [Reference]				0.87	0.34			
Yes	0.78		0.41	0.13	0.34	0.01	0.1	0.44
Number of rooms per effective household-size								
<0.35	-0.14		-0.11	0.17	0.38	0.23	0.16	0.08
0.35-0.45	-0.18		-0.1	0.4	0.49	0.47	0.39	0.25
≥0.45 [Reference]				0.43	0.50			
Irrigation-pump								
None [Reference]				0.90	0.31			
One or more	0.78	0.78	0.45	0.1	0.31	0	0.06	0.39
Radios								
None [Reference]				0.81	0.39			
≥1	0.5	0.5	0.28	0.19	0.39	0.05	0.2	0.45
Bicycles								
None [Reference]				0.62	0.49			
≥1	0.51	0.51	0.31	0.38	0.49	0.08	0.47	0.81
Rickshaw								
None [Reference]				0.90	0.30			
≥1	-0.28	-0.28	-0.15	0.1	0.3	0.16	0.06	0.03
Almirah (lockable cabinet)								
None [Reference]				0.78	0.41			
1	0.46	0.46	0.32	0.16	0.37	0.01	0.15	0.49
2		0.88	0.54	0.04	0.19	0	0.01	0.17
≥3		1.3	0.76	0.02	0.12	0	0	0.08
Wooden bed per effective household-size								
<0.2		-0.26	-0.21	0.03	0.18	0.06	0.01	0.01
0.2-0.4		-0.11	-0.11	0.19	0.39	0.3	0.1	0.12
0.4-0.6 [Reference]				0.36	0.48			
0.6-0.8		0.03	0.06	0.27	0.45	0.18	0.35	0.3
>0.8		0.14	0.13	0.15	0.36	0.06	0.2	0.23
Clock								
None [Reference]				0.46	0.50			
1		-0.12	-0.01	0.29	0.45	0.22	0.4	0.2
2		0.28	0.2	0.14	0.35	0.03	0.18	0.29
≥3		0.85	0.49	0.11	0.31	0	0.05	0.45
Television								
None [Reference]				0.91	0.28			
≥1		0.92	0.58	0.09	0.28	0	0.02	0.41
Index summaries								
% of sample used for PCA	99.7	99.8	99.5					
% of scores based on >80% of data	99.7	99.9	99.7					
% of variance explained	0.14	0.16	0.12					
No. of variables in index	15	15	30					

The last three columns give the variable means by low, middle and high groups defined as the first 40%, next 40%, and top 20%, according to their score on the Living Standards Index. All the variables are binary. The weights on indices give the change in the index in terms of standard deviations from ‘not having’ to ‘having’ the asset;

DA=Durable Assets Index;

DC=Dwelling Characteristics Index;

LSI=Living Standards Index;

PCA=Principal component analysis;

SD=Standard deviation

We created indicator variables for each level of a categorical variable. In a few instances, we merged a category with few responses into another related category. We also categorized count variables on ownership of land, durable assets, and productive assets and then created indicator variables in the same way. The PCA was performed on these indicators, except that the most common category for each variable was excluded and served as a reference.

We explored two possible ways of categorizing count variables for inclusion in the PCA. First, we used a straightforward categorization (such as 0, 1, 2-5, and >5 cattle) and used dummy variables for those categories to construct indices. One concern with this approach is that the resulting index will give a ranking of households based on total household assets without adequate adjustment for household size. This might, therefore, not be an appropriate proxy for living standards of individual household members, as households with more members would risk owning more items in a given class or category. Despite this, most previous studies using asset indices did not adjust for household size, arguing that household characteristics and many durable assets benefit the whole household, irrespective of the number of household members (1–2). Wagstaff *et al*., however, adjusted their index using the square root of the household-size (8). We adopted the idea of an effective household-size defined as ES=A+α.C where A is the number of adults, C is the number of children, and α=0.3, following the method proposed by Deaton and Paxson (11).

For our second method of categorization, we divided asset variables that could be considered household-level variables by the effective household-size before categorization. Examples of these include number of wooden beds, number of rooms in the household, and number of cattle while questions regarding the type of wall construction or presence of electricity were coded without adjustment for sample-size as before.

Before estimating the principal components, all the variables were centered at zero and scaled to have a unit variance. This way the principal component has a mean of zero, and all the variables have an effect on the principal components in proportion to the weight they are assigned by the analytical procedure.

Formally, the first principal component ‘Y’ is given by

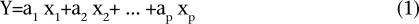

where x_1_, x_2_, …, x_p_ are the standardized variables and a_1_, a_2_, …, a_p_ are chosen to maximize the variance of ‘Y’ subjected to a_1_^2^+a_2_^2^+ … +a_p_^2^=1. Dividing the equation (1) by the standard deviation of the principal component (σ_Y_) produces a value for each household with mean ‘zero’ and variance ‘one’ which we use as our standardized index score. The standardized index score, obtained by dividing the equation (1) by the standard deviation, gives an interpretation of the coefficients. All the variables we included in the analysis are dichotomous, so a_k_/σ_Y_ gives the effect of a change from 0 to 1 (usually ‘no’ to ‘yes’, or ‘has not’ to ‘has’) on the index score. Since the index has been scaled to a unit variance, the effect of these coefficients is in units of standard deviations of the index. These coefficients are reported in Table 1 to illustrate the absolute effect of each variable on the indices [This effect is approximate because of a negligible effect of missing values on the standard deviation of the index].

Missing data were handled with a simple imputation, accepting a small bias towards the mean. This was supported by simulation studies showing that this approach did not significantly affect the ranking of households. We also performed additional simulation studies (not shown) examining various methods to correct for this bias and concluded that their marginal benefit was very low for the additional complexity.

### Ranking assets

An important practical question was faced—which are the most informative household assets to collect data on?—given limited resources to collect data. It is not immediately clear from the PCA which assets give the most information, partly because we recoded asset information into dichotomous variables. We found the following to be a reasonable measure to rank assets. It took into account the loadings given by the PCA to each of the dichotomous variables derived from the asset and weighted them by how often each loading influences the index. Formally, we defined the influence ‘I’ of an asset by

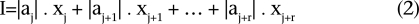

where x_j_, …, and x_j+r_ are the dummy variables used for representing each category of this asset (except the most common one, which serves as reference), and a_i_ are their loadings [if the variable only has one category included in the analysis (e.g. electricity), then r=0].

### Sub-indices

Our indices to measure living standards and wealth were based on data collected on 14 and 29 asset variables respectively. Having ranked assets in the last section, another practical question related to how many assets would researchers typically need to assess to construct a reasonably-performing index. To explore this question, we constructed sub-indices based on fewer variables, choosing 6, 9 and 12 assets to measure living standards and 8, 16 and 24 assets to measure wealth. First, we constructed the indices using the most influential assets, according to the influence measure derived earlier. The six assets used for the first sub-index, for example, are type of toilet facility, number of bicycles, type of walls, type of kitchen facility, number of clocks, and number of living-rooms, according to their ranking in Table 2. Next, we created sub-indices of the same length but chose assets at random, repeating the random selection 10 times, to establish a more plausible lower bound on the performance, in practice, of indices with fewer variables and to examine how well the measure of influence ranks the assets.

**Table 2.** Ranks of assets by influence measure on living standards index

Question	Influence
Type of toilet facility	0.11
No. of bicycles	0.11
Type of wall	0.11
Type of kitchen	0.08
No. of clocks	0.05
No. of living-rooms	0.05
No. of closets	0.05
No. of wooden cots/beds	0.05
No. of radios	0.04
Has electricity	0.04
No. of working televisions	0.03
No. of irrigation-pumps	0.03
Type of roof	0.02
No. of rickshaws	0.01

## RESULTS

### Indices constructed

Of the several indices constructed, we will focus on describing and evaluating the performance of the Living Standards Index (LSI) and, to a lesser extent, the Wealth Index (WI). The LSI, incorporating the type of material in household floor, walls, and roof and ownership of durable assets, is compatible to economic indices used in many studies seeking to measure long-term living standards (1–2). The WI incorporates, in addition to the same assets as the LSI, productive assets, such as size of land for crops, ownership of livestock, and ownership of fruit-trees or bamboo-groves. The specific variables used for the LSI are listed in Table 2 (these are also shown in Table 1, along with answer categories). Several variables were excluded from the PCA as mentioned earlier due to their non-informative nature. Of these, the source of water was excluded due to the ubiquitous nature of tubewell-use for drinking-water in this area. Ownership of motorcycle(s) was also excluded as an extremely rare reported household possession, thereby adding little to our ability to discriminate status.

**Fig. 2.**
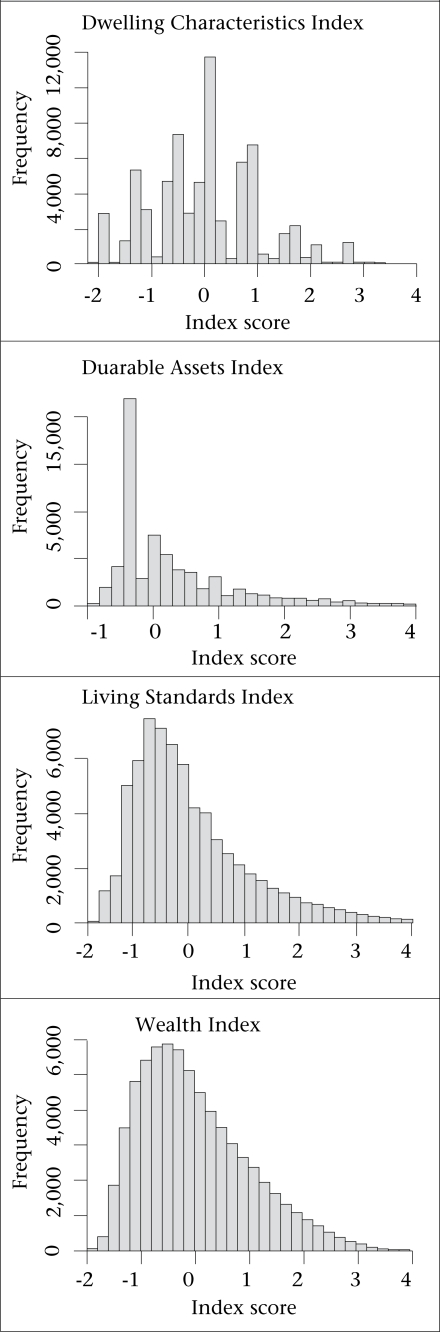
Histograms of indices

Frequent problems with PCA-based measures include clumping of the index distribution, representing clustering around a small number of values on a continuous scale, and truncation, when many households cluster in the highest or the lowest value of the distribution (1,3). Figure 2 shows that the index of dwelling characteristics exhibited some clumping and truncation but the other indices, the LSI and WI in particular, exhibited neither problem. Missing data were not a serious constraint as we had information on all assets in the LSI for 99.5% of the households.

### Household-size—to adjust or not

Results were very similar whether we adjusted for household-size or not. As discussed earlier, the concern of whether to adjust for size reflects the risk of members of larger households being erroneously assigned higher LSI scores. The indices that were adjusted for household-size showed a somewhat higher correlation to health, nutrition and demographic measures. We, therefore, preferred the adjusted indices and, in what follows, indices referred to were adjusted for household-size.

### Summary of results

Table 1 summarizes the results. The first three numeric columns show the loadings of each asset on the indices from three separate principal component analyses, adjusted by the variable and index standard deviation. These showed the effect of having an asset in terms of standard deviation distances from the index score. For example, in Table 1, moving from having no walls or walls made of branches, to tin or wood-plank walls increased the LSI by 0.3; moving from a pit-latrine to a water-sealed one increased the LSI by 0.36 [0.35-(-0.01)]; and having electricity increased it by 0.41. The next two columns give the overall mean and standard deviation of each asset variable. As binary variables, the values represent proportions of households owning each asset; for example, 41% of this population had no walls or walls made of thatch, grass, sticks, or branches, and 12% had no roof or a roof made of thatch or grass.

The last three columns give the mean of each variable by the lowest 40%, middle 40%, and the highest 20% of the LSI, as per the approach taken by Filmer *et al*. (2). The index shows a high degree of internal consistency, evident by virtually all the variables showing the trends in the mean values in the expected direction across the three strata and none showing a gradual change in an unexpected direction. (A possible exception is the downward change in ownership of rickshaw but this is consistent with findings of other studies showing such a trend for ownership of bicycle, as described by Vyas *et al*. (3), which makes intuitive sense since operating a rickshaw is a very low-paying occupation.) These last columns also allow strata of distribution of the living standards to be profiled across the asset variables. For example, a majority of those in the low LSI group (lowest 40%) live in a house with no walls or walls made of thatch, grass, sticks, or branches and have no toilet. Only 5% of this group owns a radio, 8% a bicycle, and 1% a cabinet (local term: *almirah*) that can be locked. On the other hand, the majority of those at the high end of the distribution (highest 20%) lived in houses that had tin, wood-plank, or cemented walls, and a water-sealed toilet. They had at least one lockable closet, a clock, and a bicycle; 45% owned a radio.

### Ranking assets

Rankings of assets by the influence measure are shown for the LSI in Table 2. The type of toilet ranked first, followed by the number of bicycles and the type of wall construction in the household. Table 3 compares the indices constructed with information on fewer assets chosen at random to indices where assets are chosen using this influence measure.

**Table 3.** Spearman correlations of sub-indices to original LSI and WI

Index	Using influence measure	Random (mean over 10 iterations)
Living standards		
Based on 6 assets	0.91	0.83
Based on 9 assets	0.95	0.91
Based on 12 assets	0.97	0.96
Wealth		
Based on 8 assets	0.92	0.81
Based on 16 assets	0.97	0.93
Based on 24 assets	0.99	0.98

LSI=Living Standards Index;

WI=Wealth Index

The first column of the table shows Spearman correlation coefficients calculated between each sub-index constructed from the most influential assets (based on our measure) and the corresponding index using all the assets. The high correlations indicate that the smaller indices are likely to perform similarly as predictors compared to the more complex indices and that our ranking method reliably identified the most important assets. Values in the second column of Table 3 represent the mean Spearman rank correlations between the sub-indices generated from a random selection of assets and the original indices. Notwithstanding still-high correlations, the lower values reflect some loss of association, particularly with the sub-indices with fewer than nine variables. We found indices with as few as nine assets to perform well as judged by having high correlations with the larger index and being free of truncation or clumping. Indices constructed with fewer assets showed some evidence of truncation to the left (data not shown), which would make it harder for such indices to distinguish poor from extremely poor households. This property could affect the value of using an index in predicting the demographic or health outcomes among the poor or for targeting the poorest households, e.g. to identify ultra-poor/vulnerable group programme targets.

### Asset indices as predictors of demographic and health outcomes

**Fig. 3.**
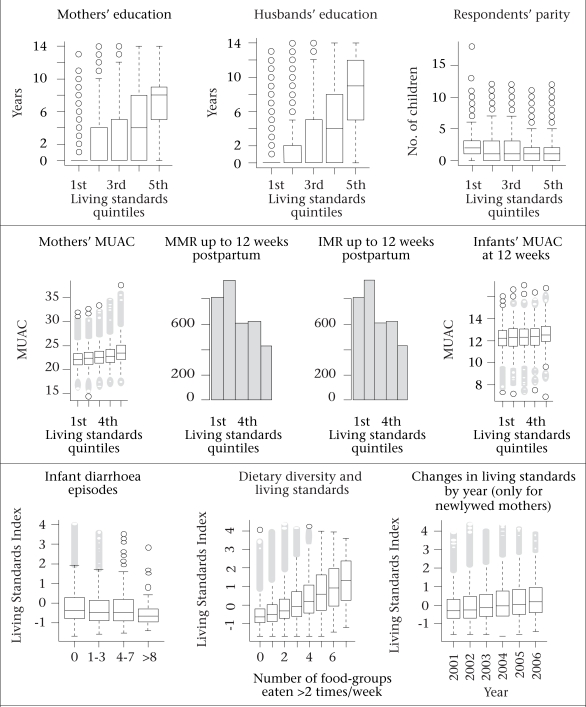
Relationships between Living Standards Index and demographic and health measures

Table 4 shows correlations among the asset indices and between indices and selected health and population measures of status and outcomes as a way of examining their predictive potential. All correlations were in expected directions, and all were nearly significantly different from zero, i.e. for all values of r≥0.03 assuming n=6,000 for lines marked with ‘*’ and r≥0.01 assuming n=50,000 for other lines. Correlation coefficients between the socioeconomic indices and health status and the outcome indicators were r≈0.17 to 0.23 for maternal and r≈0.09 to 0.13 for nutritional status of infants reflected by mid-upper arm circumference (MUAC), and r≈0.05 and r≈0.10 for maternal and infant mortality respectively. Parity negatively correlated with the SES indices. On the other hand, index correlations with maternal dietary diversity, derived from a seven-day food frequency in the first trimester of pregnancy and which would be expected to vary by social and economic well-being, were in the range of r≈0.25 to 0.35, reflecting a moderately-strong association. Figure 3 provides greater details and, specifically, the distributional details, to these relationships between the LSI and the health and demographic measures. For each association, there was a monotonic, dose-responsive and plausible relationship with the index values. Thus, education of the mother and husband rose beyond each quintile of the LSI as did the maternal dietary diversity and nutritional status (MUAC) of mothers and infants. Conversely, parity and infantile diarrhoeal episodes in the previous 12 weeks and risk of maternal and infant mortality declined with the improved LSI scores.

**Table 4.** Correlations within indices and between indices and health and population measures

Index/health or population measure	Dwelling Characteristics Index	Durable Assets Index	Living Standards Index	Wealth Index
Dwelling Characteristics Index	1			
Durable Assets Index	0.56	1		
Living Standards Index	0.89	0.84	1	
Wealth Index	0.75	0.74	0.86	1
Respondents’ years of schooling*	0.53	0.58	0.63	0.59
Husbands’ years of schooling*	0.57	0.63	0.68	0.67
Maternal MUAC	0.21	0.19	0.23	0.21
Maternal mortality before 12 weeks postpartum*	-0.066	-0.044	-0.065	-0.066
Infant's MUAC at 12 weeks after birth	0.078	0.129	0.117	0.126
No. of diarrhoea episodes among infants before 12 weeks*	-0.074	-0.037	-0.064	-0.036
Infant mortality up to 12 weeks postpartum*	-0.1	-0.11	-0.12	-0.12
Parity*	-0.094	-0.149	-0.149	-0.078
No. of food-groups eaten >2 per week*	0.29	0.31	0.34	0.35
Week of interview	0.172	0.071	0.162	0.081

Each cell is based on between 50 and 60 thousand observations, except the infant health cells which are based on between 6 and 10 thousands. All correlations are Spearman rank correlations, except those in lines marked with ‘*’, in which cases these are polyserial correlations. The food-groups counted are: meat and liver; fish; eggs; milk; dark green-leafy vegetables; other vegetables; and fruits;

MUAC=Mid-upper arm circumference

First four panels show, by quintiles of the distribution of the LSI, mothers' and husbands' years of education, number of previous livebirths, and mothers' MUAC (cm) as a proxy of nutritional status. The next two panels show boxplots of the LSI by the vital status of mothers and infants at 12 weeks after birth. The next panel shows infants' MUAC (cm) by quintiles of the distribution of the LSI. The final three panels show boxplots of the LSI by number of diarrhoea episodes of infants by 12 weeks of life, by number of food-groups, such as meat and liver, fish, eggs, milk, dark green-leafy vegetables, other vegetables, and fruits the mother eats more than twice per week, and by calendar year of study enrollment; IMR=Infant mortality rate; LSI=Living Standards Index; MMR=Maternal mortality rate; MUAC=Mid-upper arm circumference

### Rising living standards

We found a steady rise in the distribution of the LSI by the calendar year in which women were interviewed (last panel of Fig. 3). The last panel of Figure 3 shows this improvement for those women who were newly-wed women and were enrolled during the trial. This comparison is meaningful since the indices were calculated using pooled data over all years. The trial enrolled (essentially) all women in a certain geographical area soon after they were married, which suggests that this improvement was not driven by a selection effect but rather indicates a true rise in living standards, as measured by ownership of assets. This rise in living standards was also economically significant—the difference in the index scores of the average household in 2006 compared to 2001, equal to 0.5 index scores was greater than the score from having electricity (0.41), irrigation-pump (0.45), or a water-sealed or slab toilet instead of no toilet facility (0.35).

## DISCUSSION

The results showed that the indices were both internally and externally consistent, i.e. the assets were distributed as expected within low, medium, and high levels of each index, and the constructed indices correlated as expected with each other and with the health and demographic characteristics widely viewed as related to socioeconomic status respectively. The spread of each index achieved demonstrated well how this technique worked to attribute a relative ranking of socioeconomic status within a relatively-homogeneous, rural, agrarian population. Analysis of the sub-indices showed that the well-performing indices could be constructed using as few as 9-10 questions. This is an important finding to note, especially when the time and financial constraints limit the amount of subject interaction possible or the volume of data that can be collected or analyzed. It is the selection of these key variables that is, however, important when aiming at achieving parsimony without sacrificing the power to discriminate subtle levels of status in a fairly- homogeneous population.

Asset-based indices, such as those constructed here, have been used in regression models for predicting outcomes, such as school enrollments (1–2), mortality of children aged less than five years (7), and fertility (6). For this purpose, the LSI is the most conceptually appealing of those that we constructed and has the strongest associations with selected health and population measures.

Asset questions that give the most information when constructing the indices of living standards are, of course, context-specific. Those assets owned by either none or all of the households supply no information. Between those extremes, there is a continuum of how informative a particular asset is, which also depends on its direction and strength of the association with other assets in the index. Analysis of the sub-indices indicated that the shorter indices had a somewhat stronger association with a larger index when assets were chosen based on the influence measure rather than by random (Although this difference could be overstated due to capitalization on chance, it may, on balance, be understated, since particularly uninformative asset variables were excluded during early stages of the analysis).

We found that our indices, constructed using data from a large nutritional intervention community trial in rural northwest Bangladesh, were internally consistent and correlated with the health outcomes and demographic features of public-health importance as expected. This strengthens the evidence for the use of this approach in the context of rural Bangladesh and for constructing similar indices elsewhere in South Asia. The sub-indices based on assets chosen according to their influence on the original index showed that different categories and numbers of assets could supply unique information to social and economic indices. Our finding of consistently and significantly improving living standards in the area is comforting but, at the same time, leaves us without answers as to what may be bringing about this change, which may be worthy of further investigation.

## ACKNOWLEDGEMENTS

This research of the JiVitA Project was funded under the Global Research Activity (GHS A-00-03-00019-00) between the Office of Health, Infectious Diseases and Nutrition, U.S. Agency for International Development (USAID), Washington, DC, and the Department of International Health, Johns Hopkins Bloomberg School of Public Health, Baltimore, MD, USA, the Bill & Melinda Gates Foundation (Grant No. 614), Seattle, WA, USA, and the Government of Bangladesh, with additional administrative, financial or technical assistance from the USAID Mission, Dhaka, Bangladesh, the SIGHT AND LIFE Research Institute, Baltimore, MD, USA, the Canadian International Development Agency and Micronutrient Initiative (CIDA), Ottawa, Canada, and the Nutrilite Health Institute of Amway Corporation and the Access Business Group, Buena Park, CA, USA.
